# Who cares about childcare? Covid-19 and gender differences in local public spending

**DOI:** 10.1007/s10797-025-09887-8

**Published:** 2025-03-17

**Authors:** Alda Marchese, Paola Profeta, Giulia Savio

**Affiliations:** 1 Università della Svizzera italiana, Lugano, Switzerland; 2 AXA Gender Lab, Università Bocconi, Milan, Italy; 3 Department of Economics and Statistics Cognetti de Martiis, University of Turin, Turin, Italy

**Keywords:** Covid-19, Women in politics, Regression discontinuity, Local public expenditure, D72, H75, J45

## Abstract

The Covid-19 pandemic increased the salience of childcare and focused attention on the allocation of public funds for it. Focusing on Italy, one of the first countries severely impacted by the Covid-19 crisis, we analyze how male and female politicians responded to the Covid-19 pandemic in the allocation of funds to childcare. To assess causality, we analyze close mixed-gender races in Italian local elections in small municipalities without gender quotas from 2016 to 2023. Our findings show that pre Covid-19 female mayors spent more on childcare than male mayors. However, during the pandemic, the gender gap closed, as male mayors increased spending, a trend that continued in the post pandemic period.

**Supplementary Information:**

The online version contains supplementary material available at 10.1007/s10797-025-09887-8.

## Introduction

The Covid-19 pandemic increased the salience of childcare and focused attention on the allocation of public funds for it. The closure of schools and the diffusion of remote work made it clear that childcare is a policy priority. How did male and female political leaders react to this sudden shift in priorities? Do women spend more than men on childcare before, during and after Covid-19? Or, alternatively, do gender differences disappear when the salience of childcare increases?

This paper provides an empirical answer to these questions in the context of Italy, a country that was severely hit by the Covid-19 pandemic. We analyze how male and female mayors adjusted public spending on childcare during and after the pandemic. The analysis is conducted on small municipalities (fewer than 5,000 residents) where the mayor plays a relevant role in spending decisions. To assess the causal impact of the gender of the mayor on the allocation of public funds, we consider mixed-gender mayor’s elections, where a female and a male candidate compete against each other for the mayor position. Among mixed-gender elections, focusing on close races, i.e. elections that are won by a candidate with a small margin of victory over their competitor, guarantees that the gender of the mayor is as good as random (Brollo & Troiano, 2016; Gagliarducci & Paserman, 2016),^1^ and results have a causal interpretation. Using a regression discontinuity design (RDD), we show that, prior to the pandemic, the share of total spending that female mayors allocated to childcare was 38% higher than that of their male colleagues. However, during the pandemic, the gender gap closed, as male politicians increased spending on childcare, a trend that continued post pandemic. The result, which holds true after a battery of robustness checks, is consistent with a change in the salience of childcare for voters, which may induce also male mayors to care about childcare.

**Fig. 1:**
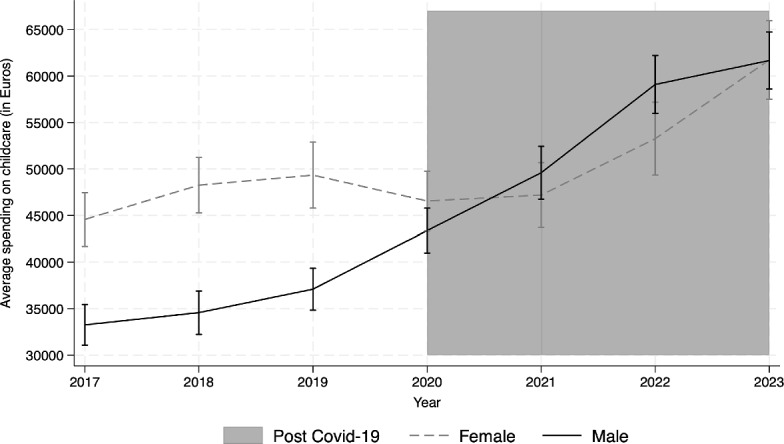
Average spending on childcare by mayor’s gender. *Notes* The figure plots the yearly average spending on childcare in Euros by gender. The dashed line represents spending in municipalities where the mayor is a woman, while the solid line represents spending in municipalities where the mayor is a man. The period under consideration is from 2017 to 2023. The shaded area represents the post Covid-19 period, which includes the during Covid-19 period (2020–2021) and the after Covid-19 period (2022–2023). 95% confidence intervals are included

The Italian context is particularly appropriate for our analysis. The province of Bergamo in northern Italy experienced one of the deadliest Covid-19 outbreaks in the world: in just one month, March 2020, 4500 people died from the virus. In Italy, more than in other countries, schools remained closed for several months during the peak of the pandemic. In May 2020 and again in March 2021, the frequency of searches for the term "Asili Nido" (day care centers for children aged 0–2) on Google Trends reached its highest level since 2013, showing a clear increase in the salience of childcare for citizens during the pandemic. The government also increased attention to childcare: a country with a structural shortage of daycare centers made significant investments during the recovery period (3108 billion), mainly financed by European resources from the Next Generation EU program within Italy’s National Recovery and Resilience Plan (PNRR). The increased salience of childcare may induce higher current expenditures of municipalities on childcare, a category of spending decided by mayors without any specific funds from the central government. Figure 1 shows trends in the level of current spending on childcare in Italian municipalities led by male and female mayors from 2017 to 2023. Consistently with the increased salience of childcare, the pandemic marks a clear turning point: after 2019, we observe an increase in childcare spending across all municipalities. Notably, municipalities led by male mayors increased their childcare spending during the pandemic, bringing their expenditure levels in line with those of municipalities led by female mayors.^2^

Our study contributes to both the literature on gender differences in childcare spending and to previous research on the consequences of the pandemic. The framework of *substantive representation*—which goes beyond *descriptive representation*—suggests that political representatives not only reflect the population’s diversity but also actively work toward the well-being of their constituents (Phillips, 1995; Mansbridge, 1999; Thomas, 1991). Gender differences in citizens’ social preferences (Chattopadhyay & Duflo, 2004; Alesina & La Ferrara, 2005; Funk & Gathmann, 2015; Clots-Figueras, 2011), the unique challenges faced by women (Berkman & O’Connor, 1993), and traditional gender roles related to family assistance and child support laws (Besley & Case, 2003), make it reasonable to theorize that female politicians are more likely to promote policies enhancing gender equality and women’s access to the labor market (see O’Brien and Rickne (2016) and the review of Hessami and da Fonseca (2020)). One major policy in this context is childcare (Bratton & Ray, 2002). However, the causal evidence from developed countries is mixed: while several studies find no significant differences between the spending decisions made by male and female politicians (Carozzi & Gago, 2023; Ferreira & Gyourko, 2014; Geys & Sørensen, 2019), others suggest that female councilors in governmental and legislative bodies are more likely than male politicians to support childcare spending (Baskaran & Hessami, 2023; Lippmann, 2019). Among this second group, a closely related study by Baskaran and Hessami (2023) provides significant insights into gender representation in Bavaria concerning childcare provision. Using RD estimations based on close mixed-gender races for the final party-specific council seats, Baskaran and Hessami (2023) demonstrate that each additional female councilor fosters the expansion of public childcare services by 40% in Bavaria. Gender differences in decision-making emerge through team dynamics: a detailed analysis of monthly council meeting records reveals that the presence of female councilors alters meeting dynamics and discussions, which results in distinct policy outcomes. Focusing on mayors, rather than councilors, we instead explore the implications of leader’s gender on policy-making. A previous study on Italy (Casarico et al., 2022) did not find gender differences in any category of spending by mayors elected in close mixed-gender races in municipalities with more than 5,000 inhabitants between 2000 and 2015 (well before the pandemic). The study however does not focus on childcare, but on general social spending.^3^ We also consider a different time span (2016–2023) to include the pandemic period and a different sample of municipalities, namely small municipalities with fewer than 5000 inhabitants, where we are able to identify the implications of the leader’s gender on policy decisions.^4^

Our study also relates to research which provides causal analysis of the different reaction of male and female leaders to the Covid-19 pandemic (Profeta, 2020). Bruce et al. (2022) develop a regression discontinuity design in closely contested mayoral races in Brazil to find that female mayors reduced deaths and hospitalizations per 100,000 inhabitants and enforced non-pharmaceutical interventions more rigorously. This highlights that female leaders performed better than their male counterparts in addressing this global challenge. Chauvin and Tricaud (2024) provide a complementary perspective for the Brazilian context, examining how these findings evolved over different phases of the pandemic. The authors observe that early on, female mayors were less likely to close non-essential businesses, leading to higher per capita deaths in their municipalities. However, as the health crisis intensified, this trend reversed. The study supports the hypothesis that female politicians tend to implement fewer containment measures when voters perceive the threat as low, while the opposite is true when voters perceive it as serious. A similar dynamic emerges in our context, where men increased childcare support to levels historically advocated by women and thus male and female politicians align their decisions about childcare when voters perceive it as a more salient issue. A different result is found by Danzer et al. (2024) who find that a higher share of female members in national governments across Europe is significantly associated with a lower likelihood of school lockdown. The paper by Danzer et al. (2024), although related to our study, does not focus on childcare spending and pursues different goals. Indeed, it adopts a cross-country perspective, while we provide a causal evaluation of the gender of the mayor on childcare spending within a single country.

The rest of the paper is organized as follows. Section 2 explains the different sources of data and the institutional context, Sect. 3 documents the empirical strategy, Sect. 4 presents the results, and Sect. 5 the conclusion.

## The institutional framework and data

### Italian municipalities

Italy comprises 20 regions, 107 provinces, and 7904 municipalities. These municipalities are split into 2369 large ones (over 5000 inhabitants) and 5535 small ones (under 5000 inhabitants). Each sub-national body has independent electoral rules. Municipal elections occur every 5 years and are staggered across municipalities. Municipal governments include a mayor, a legislative body (Consiglio Comunale), and an executive body (Giunta Comunale). All are elected simultaneously. We consider municipalities with fewer than 5000 inhabitants, where mayor candidates can be backed by only one list. The winning list receives a bonus, securing two-thirds of the council seats (majority premium), with the remaining seats distributed proportionally among other lists. Lists, formed by local parties or independent groups of citizens, can include candidates up to the number of council seats and at least three-fourths of the seats. The number of seats in councils varies by population size. The system allows semi-open lists: voters choose a party and can also vote for a specific candidate by writing the name on the ballot. Lists can be ideologically driven or locally focused (civic lists).

The mayor chairs both the Consiglio and the Giunta, holding significant policy influence. The Consiglio, a legislative and supervisory body, is publicly elected, can have up to 12 members, and approves the budget. The Giunta, whose members are chosen by the mayor among the councilors, has executive power, including proposing the budget, and comprises up to 4 members. While some types of expenditures (like healthcare) are managed by the regions, municipalities provide the majority of local public services, handling waste disposal, local transportation, social services, childcare, primary schooling, urban road maintenance, water and sewer services, environmental protection, planning, and zoning.

### Data

To investigate whether there has been a gendered response to Covid-19 in municipal spending, we focus on Italian municipalities with populations under 5000 inhabitants. This allows to concentrate on the role of the gender of the mayor, i.e. the political leader. Municipalities larger than 5000 inhabitants are subject to gender quotas and double preference voting conditioned on gender (Law 212/2012), which increase the number of female politicians in municipal bodies (Baltrunaite et al., 2019), with a potential confounding effect on the role of the gender of the mayor.^5^

Within municipalities with fewer than 5000 inhabitants, we consider those that had at least one close mixed-gender race in elections between 2016 and 2022 (Figure A.1). Our preferred sample includes a cross-section of mayors (some represented only in the pre Covid-19 period, some only during the pandemic, some only after, and some across multiple periods). However, our findings hold even when the analysis is restricted to a panel of politicians present before, during, and after the Covid-19 pandemic. We collect data on municipal elections of these municipalities from the Italian Ministry of Domestic Affairs’ official website and we merged it with data on mayor’s characteristics for the same period. Additionally, we gathered data from Open Bdap (Banca Dati Amministrazioni Pubbliche) on municipal balance sheets for the years 2016–2023.^6^ This approach ensures that mayors at various stages of their mandates are captured, avoiding the conflation of Covid-19 effects with political budget cycles, which may be gender different (Accettura & Profeta, 2021). We exclude the first budget approved by the local government after elections, as it is unclear whether this budget reflects the decisions of the newly elected officials or those of the previous administration.

Municipal revenues primarily come from taxes, tariffs, and transfers. However, there is limited flexibility in these areas, whereas municipal bodies have significant power over expenditure and budget allocation. While we provide additional evidence on entries, our main analysis focuses on municipal expenditures, divided into current and capital expenses. Capital expenses involve long-term investments in projects requiring substantial financing, whereas current expenditures cover essential services provided within the current fiscal year. In responding to the Covid-19 crisis, we hypothesize that mayors prioritized reallocating current spending over capital spending due to the immediate nature of current expenditures. Additionally, capital spending during the recovery period was heavily influenced by PNRR funds from European and national resources. As a consequence, mayors’ decisions on capital spending do not involve trade-offs with other expenditures nor reflect politicians’ priorities, making them less relevant to our research question. Our sample includes 1337 municipalities which represent around 23% of the overall population of small Italian municipalities. Total observations are more since some municipalities are repeated over time (i.e. we are often able to follow them during the entire legislature—5 years). The municipalities of our sample belong to 17 Italian regions (ordinary ones plus Sardinia and Friuli-Venezia-Giulia^7^) and 568 of them are located in the Center-South, while 769 are in the North of Italy. Table 1 shows the characteristics of the municipalities of our sample (geographic location, population, age composition of the population, education level of the population, occupation level, deaths) together with mayors and election characteristics (whether the mayor is a woman, mayor’s list, year of mandate, turnout of the election, mayor’s age and level of education and whether the mayor is the incumbent.)

**Table 1:** Summary statistics of mayor’s, election and municipality characteristics

	Obs	Mean	St. Dev.	Min	Max
North	4839	0.59	0.49	0.00	1.00
Center	4839	0.13	0.33	0.00	1.00
South	4839	0.23	0.42	0.00	1.00
Islands	4839	0.05	0.22	0.00	1.00
Special statute regions	4839	0.09	0.28	0.00	1.00
Female	4839	0.40	0.49	0.00	1.00
Civic list	4839	0.94	0.23	0.00	1.00
Right list	4839	0.03	0.16	0.00	1.00
Center list	4839	0.01	0.07	0.00	1.00
Left list	4839	0.02	0.16	0.00	1.00
Second year mandate	4839	0.30	0.46	0.00	1.00
Third year mandate	4839	0.27	0.44	0.00	1.00
Fourth year mandate	4839	0.23	0.42	0.00	1.00
Fifth year mandate	4839	0.20	0.40	0.00	1.00
Turnout	4839	0.66	0.11	0.20	0.91
Age of the mayor	4839	53	11	21	87
University Degree of the Mayor	4839	0.44	0.50	0.00	1.00
Incumbent	4839	0.41	0.49	0.00	1.00
Population	4,839	1853	1316	40	5011
Female population (share)	4839	0.50	0.02	0.30	0.58
Population aged 0-2 (share)	4839	0.02	0.01	0.00	0.05
Population aged 3-14 (share)	4839	0.09	0.02	0.00	0.18
Population aged 15-64 (share)	4839	0.62	0.04	0.37	0.76
Population aged 65+ (share)	4839	0.27	0.05	0.10	0.58
Education level (share)	4765	0.18	0.07	0.02	1.00
Employment rate	4839	0.61	0.08	0.33	0.84
Male employment rate	4839	0.36	0.04	0.17	0.61
Tourism—Hotels	4839	9	33	0	695
Average income	4839	17,791	3880	8860	37,707
Min altitude	4837	227.73	214.56	-2.00	1748
Max altitude	4837	844.04	740.71	2.00	3841
Altitude province	4837	372.78	281.65	0.00	1760
Deaths	4827	25	18	0	120
Current spending on childcare (share)	4839	0.026	0.034	0.000	0.439

This table reports descriptive statistics of municipality, election and mayor’s characteristics, namely the number of observations, mean, standard deviation, minimum and maximum values. *North*, *Center*, *South* and *Islands* are dummy variables indicating the location of the municipality. *Special* *Statute* *Regions* is a dummy equal to 1 if the municipality is in a special statute region (Sardinia and Friuli-Venezia-Giulia). *Female* is a dummy equal to one if the mayor is a woman. *Civic* *list*, *Right* *list*, *Center* *list* and *Left* *list* are dummies indicating the political affiliation of the mayor. *Second*, *Third*, *Fourth* and *Fifth* year mandate are dummies indicating the year of the electoral cycle. *Turnout* is a continuous variable indicating the share of eligible voters who cast their vote. *Age* *of* *the* *Mayor* is a continuous variable. *University* *Degree* *of* *the* *Mayor* is a dummy equal to 1 if the mayor has a university degree (or higher level of education). *Incumbent* is a dummy variable equal to 1 if the mayor was in office also during the previous electoral cycle. *Population* indicates the number of residents. *Female* *Population* indicates the share of women residing in a municipality. *Population* *aged* 0–2,3–14, 15–64 and 65+ measure the share of residents in a specific age range. *Education* *level* measures the share of enrolled people in university out of the total resident population aged 18–30. *Employment* *Rate* and *Male* *Employment* *Rate* measure the share of employed citizens out of the 15–65 years old residents, respectively for all the residents and for male ones. $Tourism-Hotels$ measures the number of hotels. *Average* *Income* is computed by dividing the total amount of declared earnings by the total number of tax payers. *Minaltitude*, *Maxaltitude* and *Altitude* *Province* are geospacial data indicating respectively the lowest and highest point of the municipality, and the altitude of the province city of the region. *Deaths* indicates the number of deaths registered. *Current* *Spending* *on* *Childcare* is the outcome variable of interest, computed dividing the amount of money (in Euros) that a given municipality allocated to childcare over the total amount of money spent

Information on municipality characteristics are retrieved from the ISTAT Database. In our sample, municipalities have on average 1,853 inhabitants. Around 2% of the population is composed by kids (age 0–2). On average, 18% of residents aged 18–30 is enrolled in university, and approximately 61% of the total population is employed. Moving on to mayors and election characteristics, around 40% of the mayors in the sample are female, 44% of them hold a university degree, and they are on average 53 years old. The vast majority of mayors -94%- belong to civic lists, and over 41% are incumbents. Average voter turnout in the elections is approximately 66%. On average, municipalities allocate 2.6% of their total expenditures on childcare, including costs related to the construction and management of nursery and preschool facilities.^8^

## Identification strategy

We rely on a sharp regression discontinuity design (RDD) to estimate the causal impact of the gender of the mayor on public spending on childcare. As standard in this design, our sample is restricted to municipalities where a mixed-gender race occurred, i.e., municipalities where a female and a male mayor candidate compete against each other in the election. We use the relative margin of victory as forcing variable in the sharp RDD. The relative margin of victory is computed as the difference in the number of votes received by the female and the male candidates relative to the number of total votes obtained by them. A positive relative margin of victory identifies municipalities where the female candidate receives more votes than the male one and she is appointed as mayor. The baseline estimated equation is the following:

1 $$\begin{aligned} \begin{aligned} SSC_{i}=&\alpha +\beta Female_{i}+\chi x_{i} + \sigma x_{i}Female_{i} + +\zeta X_{i}+\epsilon _{i} \end{aligned} \end{aligned}$$

where *SSC* refers to the share of spending on childcare in a given municipality *i*, *Female* is a dummy equal to one if the mayor is female and zero otherwise; *x* is the relative margin of victory; *X* is a vector of controls; the coefficient of the variable *Female* ($\beta$) is the RD estimator. Standard errors are clustered at municipal level. RD design allows us to estimate the average treatment effect of having a female mayor at the cutoff of zero relative margin of victory. All our results are presented for three different samples: the entire one, the sample obtained with a bandwidth of 0.50 and the sample at the optimal bandwidth, selected by one common MSE-optimal bandwidth selector (Calonico et al., 2014) around the cutoff.

Given our goal to describe gender differences as a reaction to the pandemic, we first implement the same RD design over three time periods: pre Covid-19 (2016–2019), during Covid-19 (2020–2021) and after Covid-19 (2022–2023).^9^

In order to implement such research design, two assumptions must hold: both municipalities’ distribution and characteristics of mayors, elections and municipalities must be continuous around the cutoff of the relative margin in each period. To check whether the first condition holds, we performed the McCrary test on the three periods separately and on the entire sample of our analysis (see Figure A.2 in the Appendix), verifying that the distribution of the municipalities is continuous at the cutoff. For the second assumption, we run RD regressions for municipal, election and mayor’s characteristics in the three periods separately. Results are reported in Table A1 and in Figures A.3, A.4 and A.5 in the Appendix. None of the 32 variables exhibits discontinuity across all the three periods. In the RD estimates, we include as controls the variables that are discontinuous.

As pointed out by Marshall (2024), since the effects of individual characteristics correlated to gender cannot be completely isolated, our estimates should be intended as the average effect of a bundle of characteristics associated with female mayors relative to the average bundle for male mayors in closely contested elections. As we control for all observable characteristics that are discontinuous, the concerns are limited to unobservable characteristics. In a second step, in order to verify whether RD estimates are significantly different across periods, we estimate a variation of the standard RD model, including the time dimension. We define the dummy variable ${Post}_{it}$ for a given municipality *i* in a given time *t*, equal to zero in the pre Covid-19 years (2016–2019), our baseline period, and equal to one for the years afterward. When comparing the baseline pre pandemic period with the Covid-19 one, we exclude years from 2022–2023 from the analysis. On the contrary, when comparing the baseline pre Covid-19 period with the recovery one, we exclude years 2020–2021.

We estimate the following model:

2 $$\begin{aligned} \begin{aligned} SSC_{it}=\,&\alpha +\beta Female_{it}+\gamma Post_{it} +\delta Female_{it}*Post_{it} + \chi x_{it} + \\ &+ \sigma x_{it}Female_{it} + \kappa x_{it}Post_{it}+ \rho x_{it}Female_{it}*Post_{it} +\\&+\zeta X_{it}+\epsilon _{it} \end{aligned} \end{aligned}$$

where *SSC* refers to the share of spending on childcare for a given municipality *i* in a given time *t*. As mentioned above, *Post* is a dummy equal to zero for the before Covid-19 period and one otherwise, and *Female* is a dummy equal to one if the mayor is female and zero otherwise; *x* is the relative margin of victory; *X* is a vector of controls; the coefficient of the interaction term $Post*Female$ ($\delta$) is the key parameter of interest (measuring the differential effect of female versus male mayors during and after Covid-19, relative to the pre pandemic period); the coefficient $\gamma$ represents the difference for men between the periods during/after Covid-19 and before Covid-19; the coefficient $\beta$ is the difference pre Covid-19 between female and male mayors. Standard errors are clustered at municipal level, and all our results are presented for three different samples, namely the entire one, the sample obtained with a bandwidth of 0.50 and the sample at the optimal bandwidth, selected by one common MSE-optimal bandwidth selector (Calonico et al., 2014) around the cutoff. In the estimates, we control for variables that show a discontinuity in one of the three periods. Moreover, we control for variables that, while continuous at the zero margin, vary across periods.

## Results

### Main findings

**Fig. 2:**
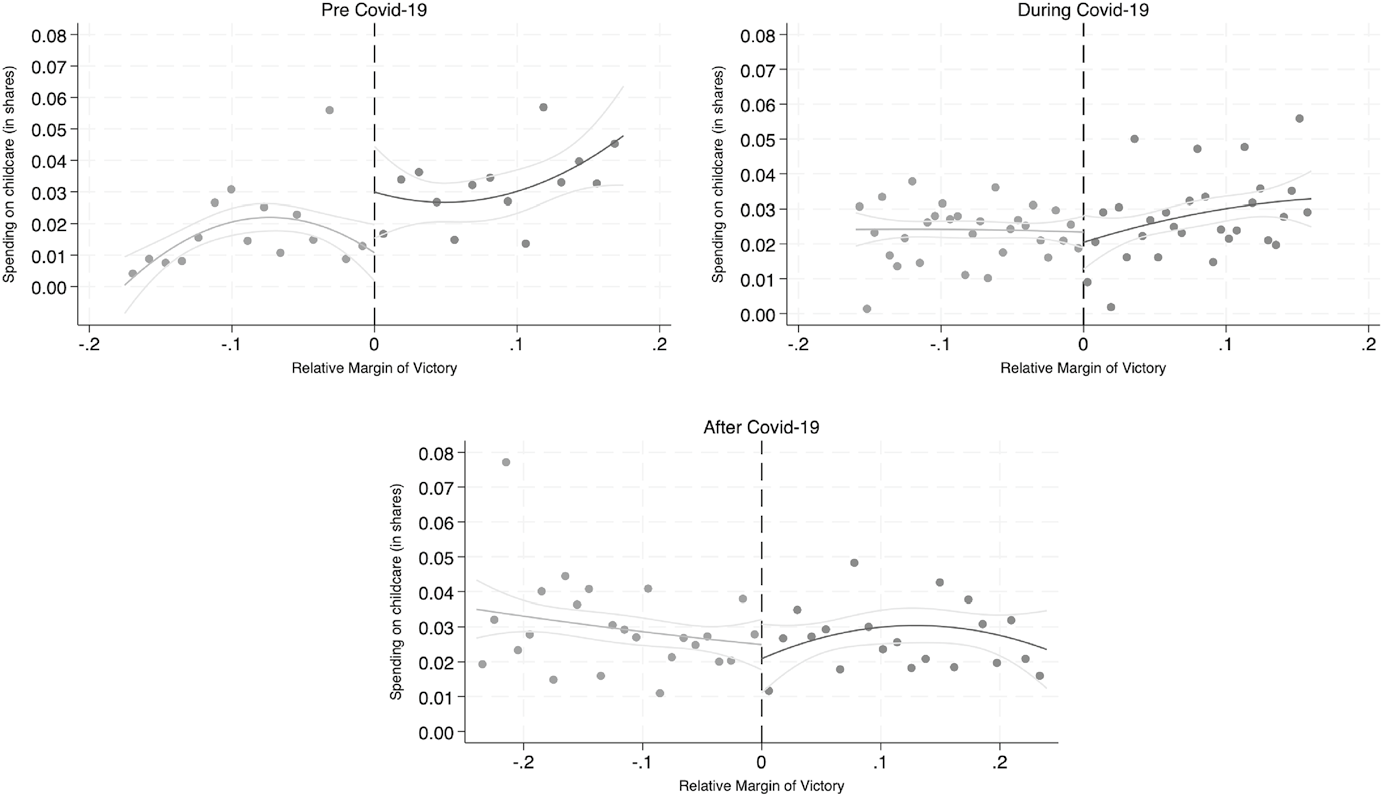
Spending on childcare (in shares). *Notes* The figure plots the binned averages of spending on childcare (in shares) in the three periods (pre from 2016 to 2019, during 2020 and 2021 and after Covid-19 2022 and 2023) separately against the relative margin of victory, together with the quadratic polynomial fit on both sides of the 0 relative margin cutoff and the 95% confidence intervals. Observations on the right-hand side (i.e. positive relative margin of victory) refer to those municipalities in which the mayor is a woman, while those on the left-hand side refer to municipalities in which the mayor is a man. The analysis is computed at the optimal bandwidth according to Calonico et al. (2014)

We begin by graphically examining whether the share of spending on childcare before, during, and after the onset of the Covid-19 pandemic is continuous at the cutoff of relative margin equal to zero. Figure 2 plots the binned averages of the share of spending on childcare at the municipal level against the relative margin of victory, together with the quadratic polynomial fit on both sides of the zero relative margin cutoff and the 95% confidence intervals. Graphs are performed including only municipalities within the optimal bandwidth around the cutoff, following Calonico et al. (2014).

As illustrated in Fig. 2, the data indicate that female mayors allocated a higher proportion of total municipal spending to childcare compared to male mayors in the pre pandemic period (2016–2019). However, this gender-specific allocation pattern is not observed during the pandemic period (2020–2021) and does not reappear in the after pandemic recovery period (2022–2023). The same pattern is also confirmed by regression discontinuity estimates performed separately for the three periods (see Table 2) in the entire sample (column 1), in a bandwidth of 0.5 around the cut-off (column 2), and at the optimal bandwidth (column 3) computed with the algorithm of Calonico et al. (2014).

**Table 2:** Spending on childcare (in shares) by period

Column	(1)	(2)	(3)
Share Childcare			
*Panel A: Pre*			
Female	0.013*** (0.00)	0.018*** (0.00)	0.009*(0.01)
Bandwidth	1.000	0.500	0.135
N	949	773	359
*Panel B: During*			
Female	0.002 (0.00)	0.001 (0.00)	− 0.005 (0.00)
Bandwidth	1.000	0.500	0.156
N	1957	1570	748
*Panel C: After*			
Female	− 0.002 (0.00)	− 0.004 (0.00)	− 0.005 (0.00)
Bandwidth	1.000	0.500	0.195
N	1933	1531	879

This table reports the results of regression discontinuity design estimation by period. The outcome variable is spending on childcare in shares. Panel A shows the results for the pre Covid-19 period (2016–2019), Panel B those of the during Covid-19 period (2020–2021) and Panel C those of the after Covid-19 period (2022–2023). *Female* is a dummy equal to 1 if the mayor is female and equal to 0 otherwise. In all specifications, we control for municipality, election, and mayor’s characteristics, which are not continuous at the threshold. More specifically, in Panel A we control for the dummies south, islands, and special statute regions, for the share of female population, and for altitude province dummy. In Panel B, we control for the dummy third year of mandate, education of the mayor, and age of the mayor. In Panel C, we control for education of the mayor, islands dummy, employment rate, and male employment rate. Results are carried out at three different bandwidths: whole sample (column 1), 0.5 (column 2), and optimal bandwidth according to Calonico et al. (2014) (column 3). *$p<$ 0.10, **$p<$ 0.05, ***$p<$ 0.01

To rigorously compare regression discontinuities across different periods, we then estimate equation 2. The results are presented in Table 3. Columns 1–3 include data from 2016 to 2021, allowing for a comparison of gender differences in spending before Covid-19 (2016–2019) with those during the pandemic (2020–2021). Columns 4–6 encompass data from the pre pandemic period (2016–2019) and the recovery period (2022–2023), facilitating a comparison of gender differences in spending before Covid-19 with those following the end of the Covid-19 emergency. Different columns include different samples, namely the entire one (columns 1,4), only municipalities in a bandwidth of 0.5 around the cutoff (columns 2,5), and only municipalities in the optimal bandwidth around the cutoff (columns 3,6) computed with the algorithm of Calonico et al. (2014). The main coefficient of interest is $\delta$, capturing the differences between female and male mayors across different periods.

**Table 3:** Current spending on childcare (in shares) over time

Period	Pre(2016–2019)–During (2020–2021)			Pre (2016–2019)–After(2022–2023)		
Column	(1)	(2)	(3)	(4)	(5)	(6)
*Share childcare*						
Female	0.009** (0.00)	0.014*** (0.01)	0.019* (0.01)	0.010** (0.00)	0.015*** (0.01)	0.018 (0.01)*
Post	0.003 (0.00)	0.007** (0.00)	0.016** (0.01)	0.007** (0.00)	0.011*** (0.00)	0.020** (0.01)
Post*Female	− 0.007 (0.00)	− ** (0.01)	− (0.01)	− (0.01)	− (0.01)	− (0.01)
Female+Post*Female	0.003	0.002	− 0.002	− 0.001	− 0.001	− 0.008
Post+Post*Female	− 0.003	− 0.005	− 0.005	− 0.004	− 0.006	− 0.007
Bandwidth	1.00	0.50	0.11	1.00	0.50	0.12
$\hbox {R}^2$	0.173	0.183	0.203	0.166	0.175	0.204
N	2863	2318	855	2841	2281	843

The table compares RD estimates over time. The outcome variable is spending on childcare in shares. Columns 1–3 include data from 2016 to 2021, allowing for a comparison of gender differences before Covid-19 (2016–2019) with those during the pandemic (2020–2021). Columns 4–6 include data from the pre pandemic period (2016–2019) and the recovery period (2022–2023), allowing for a comparison of gender differences before Covid-19 with those following the end of the Covid-19 emergency. *Female* is a dummy equal to 1 if the mayor is female and equal to 0 otherwise; *Post* is a dummy that identifies time. In columns 1–3, it is equal to 1 if the observation is in the during Covid-19 period and 0 for the pre Covid-19 period. In columns 4–6, it is equal to 1 if the observation is in the after Covid-19 period and 0 for the pre Covid-19 period. $Post*Treatment$ is the variable of interest. Results for the pre-during analysis (1–3) and the pre-after analysis (4–6) are carried out at three different bandwidths: whole sample (columns 1 and 4), 0.5 (columns 2 and 5), and optimal bandwidth according to Calonico et al. (2014) (columns 3 and 6). In all specifications, we control for municipality, election, and mayor’s characteristics, which are not consistently continuous in the three periods and for covariates that are not similar across periods. Therefore, in columns 1–3, we control for turnout, special statute regions, average income, altitude max, altitude province, south, islands, north, employment rate, male employment rate, second year of mandate, third year of mandate, fourth year of mandate, fifth year of mandate, share of population aged 0–2, share of population aged 15–64, share of population aged 65+, share of female population, education of the mayor, age of the mayor and average education level of the municipality, and in columns 4–6, we control for second year of mandate, third year of mandate, fourth year of mandate, fifth year of mandate, turnout, education of the mayor, age of the mayor, center list, south, islands, north, special statute regions, average income, share of female population, share of population aged 0–2, share of population aged 3–14, share of population aged 15–64, share of population aged 65+, employment rate, male employment rate, altitude province and average education level of the municipality. Standard errors, reported in parentheses, are clustered at the municipal level. *$p<$ 0.10, **$p<$0 .05, ***$p<$ 0.01

If we compare the pre Covid-19 period with the years of the pandemic (columns 1–3), we document three facts. (i) Consistently across specifications, before the pandemic, female mayors were allocating to childcare a higher fraction of total spending compared to males, namely from 1 up to 1.9 percentage points (pp) more, which translates into a difference of 38%.^10^ (ii) After the pandemic, male mayors increased their share of spending on childcare. Although the effect is not robust for the entire sample, the coefficient of the variable *Post* is significant at the 5% level in the restricted samples. Point estimates range from 0.007 to 0.016. (iii) As a result of the increase in spending of male mayors, the interaction term $Post*Female$ turns negative and significant in two out of three specifications, suggesting that the difference in spending between female and male mayors shrinks during Covid-19, compared to the years before. Is this new equilibrium stable in the recovery years? In columns 3–6, we compare the pre-Covid-19 gender differences to those in the recovery period. Again, although women were initially spending more than men, we document an increase in male politicians’ spending in the recovery period, compared to 2016–2019, with an interaction term ranging from $-$0.011 to $-$0.026.

### Explaining the result: the salience of childcare

In this section we provide evidence that our main result is consistent with an increased salience of childcare during the pandemic among citizens, which is reflected by mayors’ choices. Prolonged lockdown measures, the rise of remote work, and limited access to schools may have increased the salience of childcare and influenced public opinion on the relevance of childcare expenditures. Male parents, in particular, experienced the daily burden of household organization and childcare management-tasks typically shouldered by women-spending more time than usual on these activities (Del Boca et al., 2020; Sevilla & Smith, 2020; Farré et al., 2022; Angelici & Savio, 2024). In absence of direct evidence which could support this argument, we draw suggestive and consistent insights from different heterogeneity analyses.

First, we investigate whether the change in childcare spending by male mayors is more pronounced in municipalities where the need for childcare became more urgent during the pandemic, such as in areas where schools remained closed for a longer period of time. While in 2020, lockdown measures-including school closures-were uniformly implemented across Italy, in 2021, school closures were determined at the regional level using an algorithm based on hospitalization rates, fatalities, and hospital capacity. This algorithm, computed by the central emergency management, relied on non-publicly available data and was difficult to predict for experts, citizens, and local leaders alike. We thus differentiate between municipalities in regions that experienced a number of school closure days above and below the median (the median is 89 days). Specifically, we focus on closures affecting nurseries and kindergartens, excluding weekends, as well as local and national holidays. Results are reported in Table 4.

**Table 4:** School closures

N. of days school closures	Above median			Below median		
Column	(1)	(2)	(3)	(4)	(5)	(6)
Share Childcare						
Female	0.008 (0.01)	0.020*** (0.01)	0.006(0.01)	0.011 ** (0.01)	0.009 (0.01)	− 0.001 (0.01)
Post	0.008 (0.01)	0.015** (0.01)	0.015** (0.01)	0.006 (0.00)	0.008 (0.01)	0.001 (0.01)
Post*Female	− 0.008 (0.01)	$$-0.018{**}$$ (0.01)	− 0.010(0.01)	$$-0.012{*}$$(0.01)	$$-0.017{*}$$(0.01)	− 0.004(0.01)
Female+Post*Female	− 0.000	0.002	− 0.004	− 0.001	− 0.007	− 0.005
Post+Post*Female	0.000	− 0.003	0.005	− 0.006	− 0.008	− 0.003
Bandwidth	1.00	0.50	0.15	1.00	0.50	0.18
$\hbox {R}^2$	0.148	0.170	0.260	0.160	0.167	0.179
N	1542	1262	592	1299	1019	575

The table compares RD estimates over time for municipalities in which schools have been closed in the years 2020–2021 more days than the median (columns 1–3) and less days than the median (columns 4–6). The analysis is carried out only on the period pre-after Covid-19. In all columns, the outcome variable is spending on childcare in shares. *Female* is a dummy equal to 1 if the mayor is female and equal to 0 otherwise; *Post* is a dummy equal to 1 if the observation is in the after Covid-19 period and 0 for the pre Covid-19 period. Observations from the years 2020 and 2021 are not included in this analysis. $Post*Treatment$ is the variable of interest and it identifies female vs male after Covid-19, compared to female vs male before Covid-19. Results are carried out at three different bandwidths: whole sample (columns 1 and 4), 0.5 (columns 2 and 5), and optimal bandwidth according to Calonico et al. (2014) (columns 3 and 6). In all specifications, we control for municipality, election, and mayor’s characteristics, which are not consistently continuous in the three periods and for covariates that are not similar across periods, namely second year of mandate, third year of mandate, fourth year of mandate, fifth year of mandate, turnout, education of the mayor, age of the mayor, center list, south, islands, north, special statute regions, average income, share of female population, share of population aged 0–2, share of population aged 3–14, share of population aged 15–64, share of population aged 65+, average education level of the municipality, employment rate, male employment rate, altitude province. Standard errors, reported in parentheses, are clustered at the municipal level. *$p<$ 0.10, **$p<$ 0.05, ***$p<$ 0.01

The table shows that the increased childcare support by male mayors over time (captured by the coefficient of the variable *Post*) is higher in regions experiencing more school closures during the pandemic.^11^

We then examine whether the increase in male mayors’ spending is more pronounced among mayors with re-election incentives (compared to non re-electable ones), as these politicians are more likely to respond to voters’ mandates.^12^ We compare gender differences in spending after the Covid-19 pandemic with those before the crisis, distinguishing between mayors serving their final term (columns 1–3) and those eligible for re-election (columns 4–6). Findings are shown in Table 5.

**Table 5:** Re-election incentives

Mayor	Cannot be reelected			Can be reelected		
Column	(1)	(2)	(3)	(4)	(5)	(6)
Share Childcare						
Female	0.027 (0.02)	0.077*** (0.02)	0.065**(0.03)	0.009**(0.00)	0.010*(0.01)	0.004(0.01)
Post	0.001 (0.01)	0.021 (0.02)	− 0.011 (0.03)	0.010** (0.00)	0.015*** (0.00)	0.016*** (0.01)
Post*Female	− 0.030(0.02)	$$-0.086{***}$$ (0.02)	$$-0.074{**}$$ (0.03)	− 0.008 (0.01)	$$-0.013^{*}$$ (0.01)	$$-0.015^{*}$$ (0.01)
Female+Post*Female	− 0.003	− 0.009	− 0.009	0.001	− 0.004	$-0.010{*}$
Post+Post*Female	− 0.030	$$-0.065{***}$$	$$-0.086{***}$$	0.002	0.002	0.001
Bandwidth	1.00	0.50	0.19	1.00	0.50	0.16
$\hbox {R}^2$	0.183	0.253	0.510	0.190	0.208	0.215
N	396	274	137	1801	1505	790

The table compares RD estimates over time for mayors who cannot be reelected (columns 1–3) and for those who can be reelected (columns 4–6). We include only mayors elected in the pre Covid-19 period (i.e. before 2020). The analysis is carried out only on the period pre-after Covid-19. In all 6 columns the dependent variable is spending on childcare in shares. *Female* is a dummy equal to 1 if the mayor is female and equal to 0 otherwise; *Post* is a dummy equal to 1 if the observation is in the after Covid-19 period and 0 for the pre-Covid-19 period. Observations from the years 2020 and 2021 are not included in this analysis. $Post*Treatment$ is the variable of interest and it identifies female vs male after Covid-19, compared to female vs male before Covid-19. Results are carried out at three different bandwidths: whole sample (columns 1 and 4), 0.5 (columns 2 and 5), and optimal bandwidth according to Calonico et al. (2014) (columns 3 and 6). In all specifications, we control for municipality, election, and mayor’s characteristics, which are not consistently continuous in the three periods and for covariates that are not similar across periods, namely second year of mandate, third year of mandate, fourth year of mandate, fifth year of mandate, turnout, education of the mayor, age of the mayor, center list, south, islands, north, special statute regions, average income, share of female population, share of population aged 0–2, share of population aged 3–14, share of population aged 15–64, share of population aged 65+, average education level of the municipality, employment rate, male employment rate, altitude province. Standard errors, reported in parentheses, are clustered at the municipal level. *p< 0.10, **p< 0.05, ***p< 0.01

The table shows that the increase of childcare spending by male mayors is driven by re-electable mayors, and it amounts to 1/1.6 pp. Female mayors do not exhibit a similar increase in spending.^13^ These trends suggest that the increase in childcare spending among male politicians is driven by those leaders facing re-election incentives, suggesting that their budgetary decisions may reflect voters’ preferences.

Interestingly, male politicians might be responding because childcare is perceived as a more gender-neutral priority in the aftermath of the Covid-19 pandemic, rather than a female issue. Future studies using data on preferences of male and female citizens before and after the pandemic are needed to confirm this argument.

### Robustness checks

The results survive to a series of robustness checks. In Table A.2 we replicate the main results for specific subsample, and we rely on alternative RD model specifications. In particular, in columns 1–2, we narrow our sample to a panel of mayors who were in office before, during, and after the pandemic, confirming that our findings hold also for a balanced dataset. In columns 3–4, we rely on a second-order polynomial regression, while the main analysis rely on a linear fit. In columns 5–6, we focus on mayors elected before the pandemic to ensure that our results are not influenced by a different group of politicians elected after the spread of the pandemic. Finally, in columns 7–8 we report estimates without controls.^14^

Second, we check that our results are overall not sensitive to the choice of specific bandwidths (Figure A.6). Third, we graphically present the RD estimates separately for different years (Figure A.7) and we replicate our analysis by excluding one year at a time (Table A.3). Fourth, we rely on alternative definitions of the time dummies. Initially, we pool during Covid-19 and after Covid-19 years (defining a dummy *Post* equal to one for the entire period 2020–2023, and zero otherwise) and we compare them to the pre pandemic years. As shown in Table A.4, this does not affect our results. Next, we run a model that includes data for all periods, interacting the *Female* dummy with two time dummies: the first, *t*1, equals one before 2020 and zero otherwise, and the second, *t*2, equals one for the post Covid-19 years (after 2021) and zero otherwise. Results displayed in Table A.5 rely on the following model:

3 $$\begin{aligned} \begin{aligned} SSC_{it}=\,&\alpha +\beta Female_{it}+\gamma t1_{it} +\delta Female_{it}*t1_{it} + \chi x_{it} + \\ &+\sigma x_{it}*Female_{it} + \kappa x_{it}*t1_{it}+ \tau x_{it}*Female_{it}*t1_{it} +\\ &+\gamma t2_{it} +\eta Female_{it}*t2_{it} + \psi x_{it} + \\ &+\omega x_{it}*Female_{it} + \tau x_{it}*t2_{it}+ \rho x_{it}*Female_{it}*t2_{it} +\\&+\zeta X_{it}+\epsilon _{it} \end{aligned} \end{aligned}$$

The coefficient of the interaction term $Female*t1$ captures the difference between the gender gap before the pandemic and the gender gap in the Covid-19 years. Since the estimated coefficient is positive and significant in the local estimates, we conclude that the gender difference existing before the pandemic shrinks in the two years of the Covid-19 period (2020–2021). This pattern is mainly driven by male politicians increasing support for childcare, as suggested by the coefficient of *t*1 (negative and significant), which measures the share allocated to childcare by male mayors before the pandemic, compared to during Covid-19. Female politicians, instead, do not change the share of spending allocated to childcare over time, as signaled by the coefficient of the combination $-t1-Female*t1$. Hence, we conclude that this alternative specification confirms the dynamic described in Table 3.

Fourth, we exclude the possibility that the closing of the gap is due to a higher representation of women (after the pandemic) among councilors in municipalities led by male mayors, which might have exerted pressure on them to increase childcare spending. Table A.6 shows that the interaction term remains similar for municipalities with a number of female councilors below or above the median^15^ suggesting that the increase in childcare support driven by male mayors does not appear to be attributable to more female-dominated councils.

Finally, we address the concern that the mayor’s gender may be correlated with other characteristics, particularly political ideology. Although we ensure that party ideology is continuous at the zero relative margin cutoff, political competition in our sample of municipalities predominantly involves civic lists. These are parties without formal political ideologies, claiming instead to have a special connection to the local area and to prioritize its interests over ideological concerns. However, it is possible that these mayors, despite identifying with civic lists, hold ideological views, and that mayors of different genders may tend to have different ideological perspectives. If true, the differences attributed to female mayors might simply reflect more left-wing (or right-wing) positions. To address this possibility, we conduct two checks. First, we verify that in larger municipalities, where civic lists are less prevalent, the likelihood of being left-wing or right-wing is similar for both female and male mayors (Fig. A.8). Second, we examine various categories of social spending that the literature typically associates with either left-wing or right-wing politicians (Potrafke, 2017). We replicate our main analysis for spending on youth, social rights, and health (typically left-wing), as well as spending on law and order (typically right-wing). As shown in Table A.7 (columns 1–4), in none of these categories the coefficient of *Female* replicates the effect observed when childcare spending is the outcome of interest. These tests suggest that our findings are not merely a reflection of ideological differences between female and male mayors.

### Other expenditures and revenues

We have documented an increase in allocation of funds to childcare by male mayors after Covid-19. In Table A.7 we provide evidence about other expenditures and revenues. Columns 1–8 analyze all other categories of spending, namely expenditures on youth, law and order, social rights, health, transportation services and urban planning, institutions, debt, and support for economic activities. Column 9 reports total spending and columns 10–12 consider different types of local revenues: tax revenues, non-tax revenues (such as fine, rental income, and proceeds from the sale of goods and services), and transfer revenues (including contributions from businesses, households, institutions, and higher levels of government). As discussed in the previous section, the arrival of Covid-19 did not lead to gendered changes in spending on traditionally left-wing expenditures (columns 1–4). Regarding other spending categories, we observe an increase in debt alongside a decline in transportation services in the post Covid-19 period compared to pre Covid-19 years. However, these trends are consistent across municipalities led by both female and male mayors. Thus, the significant increase in the share of childcare spending by male mayors is funded through a general reduction in expenditures across various categories, without a single category being significantly impacted. Column 9 shows that male mayors spent more overall than female mayors during and after the pandemic compared to before and receive greater overall revenues (column 13), driven by an increase in transfer revenues, at least during the Covid-19 years (Panel A).

### Estimated effect

Our findings show that before the pandemic female mayors allocate a larger share of the total budget to childcare, about 1 pp more than male mayors. On average, municipalities were spending 2% of their total budget^16^ on childcare before the crisis, so an increase of 1 pp represents an additional 15,000 Euros. How sizable is this effect? Using some basic assumptions, we can perform a rough calculation. A full-time gross teacher’s salary is around 22,000 Euros,^17^ and the teacher-to-pupil ratio in kindergartens is on average 1:8.^18^ Additionally, the number of children aged 6–24 months is about 2% of the total population. In our sample of small municipalities, with an average population of 1800, this means there are around 36 children in this age group per municipality. The additional 15,000 Euros allocated by female mayors-roughly 65% of a teacher’s annual salary-could expand kindergarten capacity by 5 seats (out of 36), which represents 13% of the total demand. It’s important to note that this 13% is a lower-bound estimate, as not all children in this age group may attend kindergarten.

## Conclusion

In the context of small Italian municipalities before Covid-19, we show that being a female mayor leads to a higher spending on childcare. However, following the increased salience of childcare for voters during the pandemic, male mayors increase their spending on childcare up to the level of female mayors. Our interpretation of the findings is that changes in the socio-economic context, such as crises like the Covid-19 pandemic, may modify voters’ priorities, which in turn are reflected in public spending decisions. The identity of leaders, in particular their gender, becomes less relevant under these changing conditions. Future work will evaluate the economic impact of expanding childcare services in response to citizens’ socio-economic needs, such as accessibility, equality and overall benefits for the society.

## Supplementary Information

Below is the link to the electronic supplementary material.

Supplementary file 1 (pdf 14237 KB)

## Data Availability

Information on municipality characteristics are retrieved from the ISTAT Database: https://esploradati.istat.it/databrowser/#/. Data on municipal balance sheets are retrieved from Open Bdap (Banca Dati Amministrazioni Pubbliche): https://openbdap.rgs.mef.gov.it. We collect data on municipal elections of these municipalities from the Italian Ministry of Domestic Affairs’ official website and merged it with data on mayors’ characteristics: https://elezioni.interno.gov.it. Data on school closures has been collected by hand from newspapers and national decree.

## Footnotes

1 We are aware of the concerns raised by Marshall (2024) on the use of close races to identify individual characteristics, such as gender, which we will discuss in Sect. 3.

2 A similar graph is obtained if spending on childcare is divided by the share of the population in the 0–2 age range.

3 Our use of a different database on public spending allows us to consider spending on childcare items separately from general social spending.

4 As detailed in Sect. 2, municipalities larger than 5000 inhabitants are subject to affirmative action regulations which may create a confounding setting. Moreover, in our sample of municipalities with fewer than 5000 inhabitants, the *Giunta Comunale* and the *Consiglio Comunale* are relatively small bodies, making the mayor’s decisions likely to have a greater impact.

5 On gender quotas, female mayors and women in municipal councilors see Spaziani (2022).

6 We start our analysis in 2016, as starting from this year municipalities with fewer than 5000 inhabitants are not subject to the Stability Pact. We include final budgets for the years 2016–2022 and provisional budgets for the year 2023, as the final ones are not yet available.

7 We had to exclude Sicily from our analysis since political level data were not available for this region.

8 In the data provided by the municipalities, there are no disaggregated records available that document specific costs (e.g.,related to personnel, building maintenance, utilities, educational and recreational materials).

9 These periods are defined based on the timeline of the state of emergency declared by the Italian government, which was first issued on January 31, 2020, and extended multiple times. The emergency officially ended with Decree Law No. 24 on March 24, 2022. Therefore, we classify the entire years of 2020 and 2021 as the Covid-19 period, and 2022 and 2023 as the after Covid-19 period.

10 38% is a conservative estimate, based on the lower value for *Post* (0.009) and the average share of expenditures allocated to childcare (0.026).

11 Note that we do not include observations of the pandemic years in this analysis, because other urgent needs during that time might have influenced politicians’ responses differently, such as focusing on cost savings associated with school closures.

12 In municipalities with fewer than 3000 inhabitants mayors can be re-elected maximum 3 times in a row, while in municipalities with 3000–5000 inhabitants mayors can be re-elected maximum 2 times in a row.

13 Male mayors non re-electable do not significantly change childcare spending, while women seem to decrease it. This produces a reduction of the gender gap also among non re-electable mayors, but driven by women, probably because they were already allocating a higher proportion of spending to childcare compared to their male counterparts before the pandemic and they had to reallocate funds to other priorities during the pandemic.

14 In the balanced dataset we include only municipalities for which we have at least one observation per period (i.e. pre, during, after). Instead, for the robustness check including only mayors elected in the pre Covid-19 period we consider all municipalities for which the mayor has been elected before the pandemic. Therefore, we allow for municipalities observed only in one or two periods as long as the election took place before 2020. For these reasons, the sample size in columns 1–2 is different from that of columns 5–6.

15 The median value of the share of female members in local government is 0.33.

16 The total annual budget of municipalities in our sample is around 1,500,000 Euros.

17 The salary, set at the national level, is the same across different regions. Source: Contratto collettivo nazionale del lavoro delle scuole dell’infanzia e servizi educativi.

18 The teacher-to-pupils ratio depends on the age of the children and it is decided at regional level.
