# Supplementary material for: Who cares about childcare? Covid-19 and gender differences in local public spending
Source: Int Tax Public Financ. 2025 Mar 17;32(6):1990–2010. doi: 10.1007/s10797-025-09887-8 (PMC12634775; doi:10.1007/s10797-025-09887-8)
Supplement: Supplementary file 1 — (pdf 14237 KB) [file 10797_2025_9887_MOESM1_ESM.pdf]

A Online Appendix

Figure A.1: Map of Italian municipalities

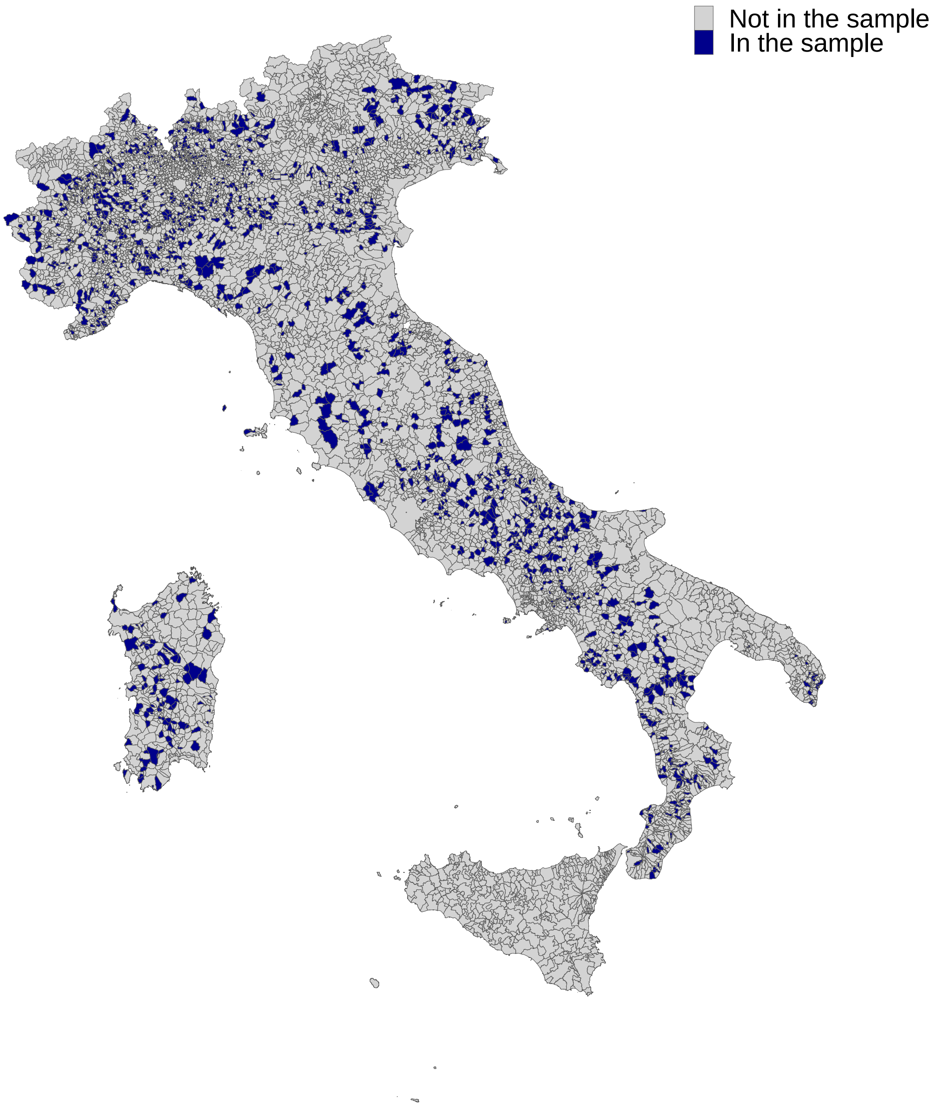

Notes: This figure displays a map of Italian municipalities. Municipalities included in our sample, which had a mixed election between 2016 and 2022 and have fewer than 5,000 inhabitants, are shaded in dark blue. All other municipalities, not included in our sample, are shaded in light grey.

Figure A.2: McCrary test

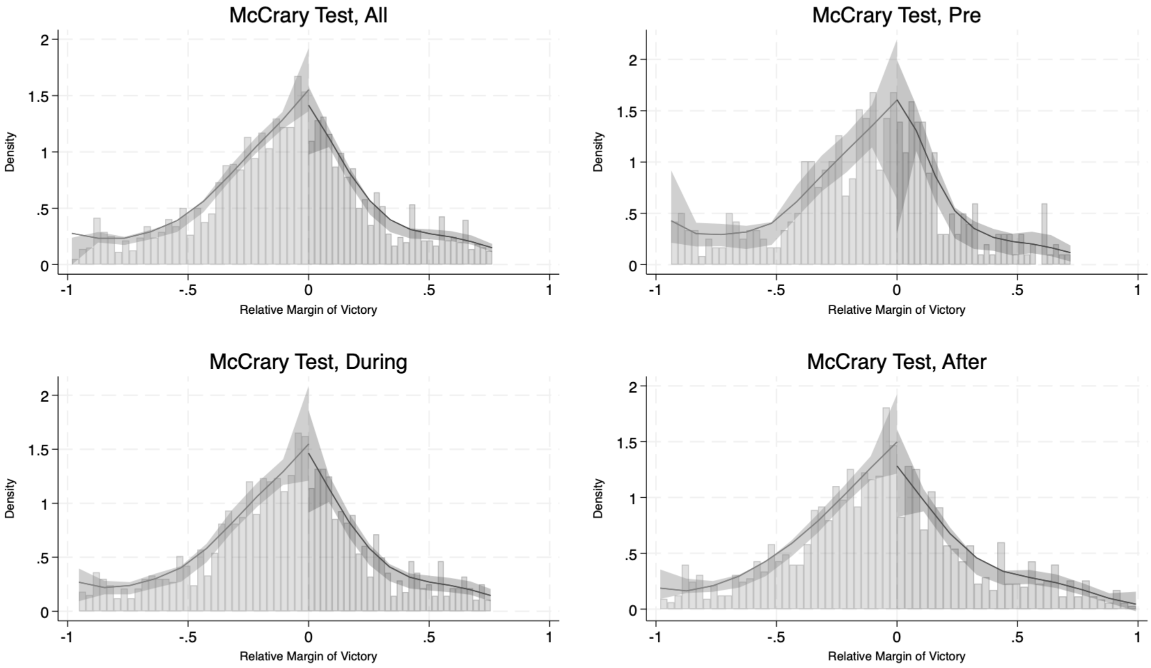

Notes: The four figures plot the density of mixed gender close races, together with the 95% confidence intervals. Figures are provided for the entire sample for the time period of the analysis (2016-2023) and for the three periods of analysis separately. The sample includes municipalities with fewer than 5,000 residents that held elections in the period 2016-2022. Observations on the right hand side (i.e. positive relative margin) refer to those municipalities in which the mayor is a woman, while those on the left hand side refer to municipalities in which the mayor is a man.

Figure A.3: Mayor's, election and municipality characteristics Pre Covid-19

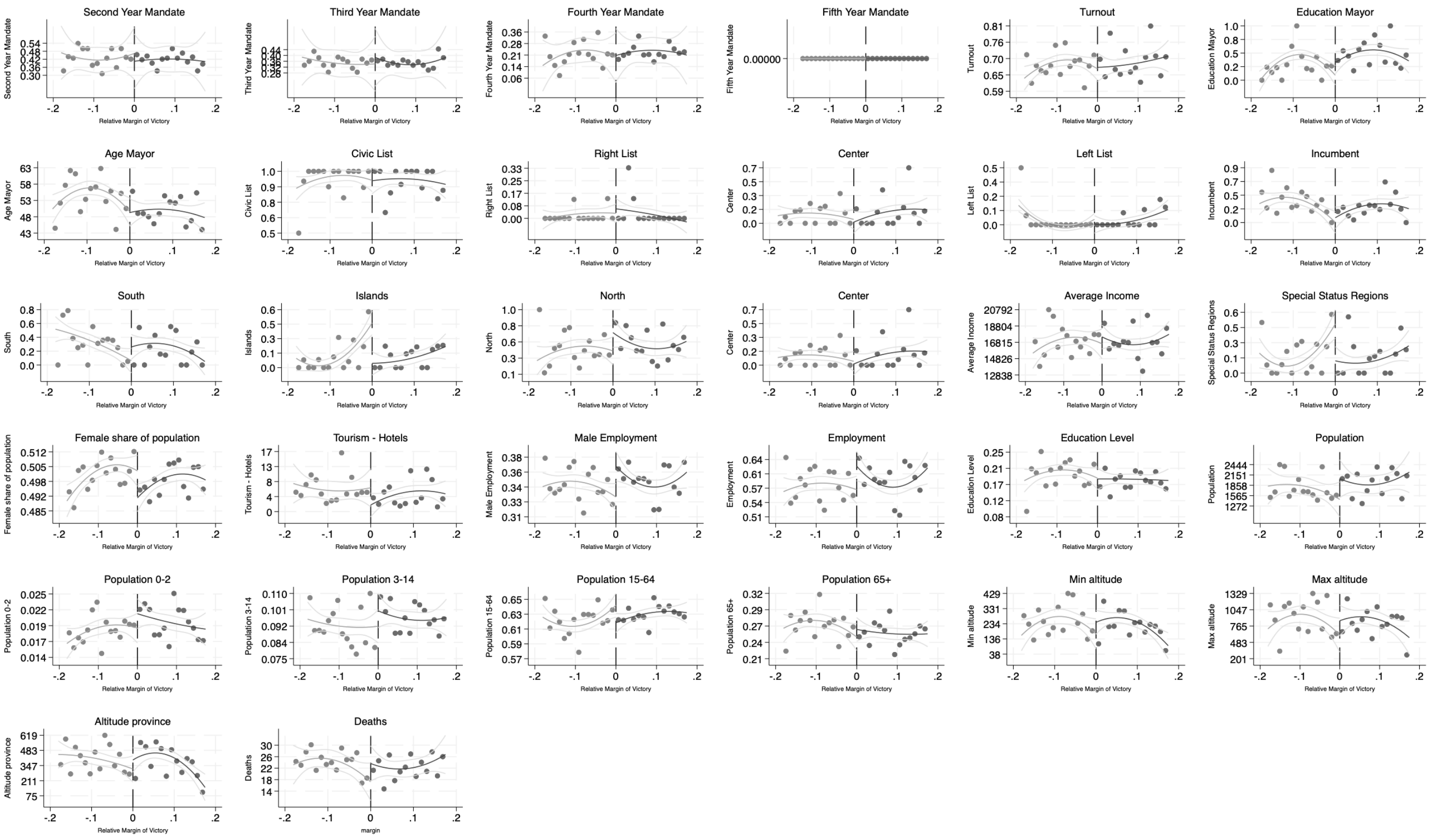

Notes: The figure plots the binned averages of 32 variables for mayor, election and municipalities characteristics against the relative margin, together with the quadratic polynomial fit on both sides of the 0 relative margin cut-off and the 95% confidence intervals. These plots refer only to the pre Covid-19 period (2020-2021). Observations on the right hand side of the cut-off refer to those municipalities in which the mayor is a woman, while those on the left hand side refer to municipalities in which the mayor is a man.

Figure A.4: Mayor's, election and municipality characteristics During Covid-19

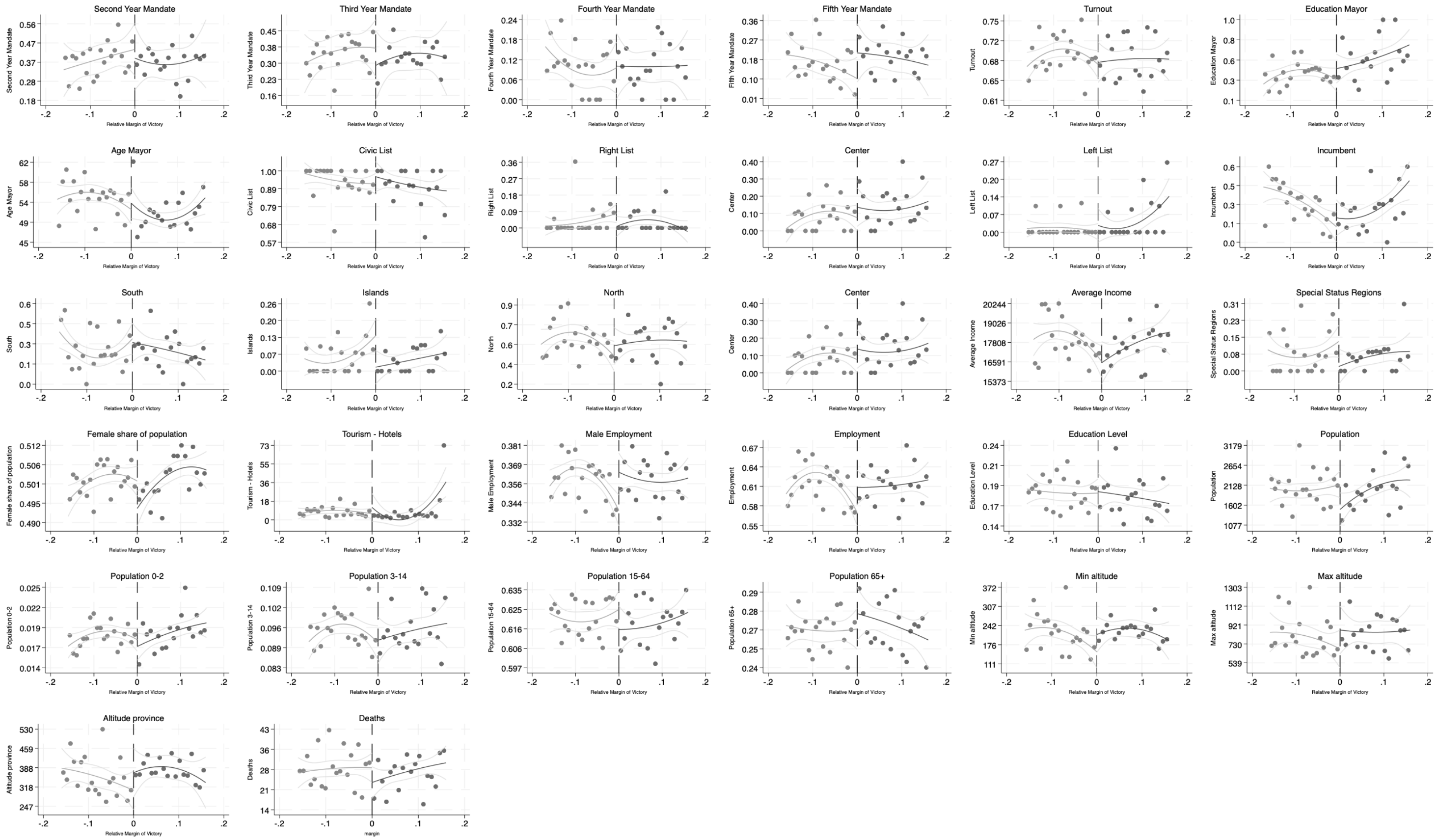

Notes: The figure plots the binned averages of 32 variables for mayor, election and municipalities characteristics against the relative margin, together with the quadratic polynomial fit on both sides of the 0 relative margin cut-off and the 95% confidence intervals. These plots refer only to the during Covid-19 period (2022-2023). Observations on the right hand side of the cut-off refer to those municipalities in which the mayor is a woman, while those on the left hand side refer to municipalities in which the mayor is a man.

Figure A.5: Mayor's, election and municipality characteristics After Covid-19

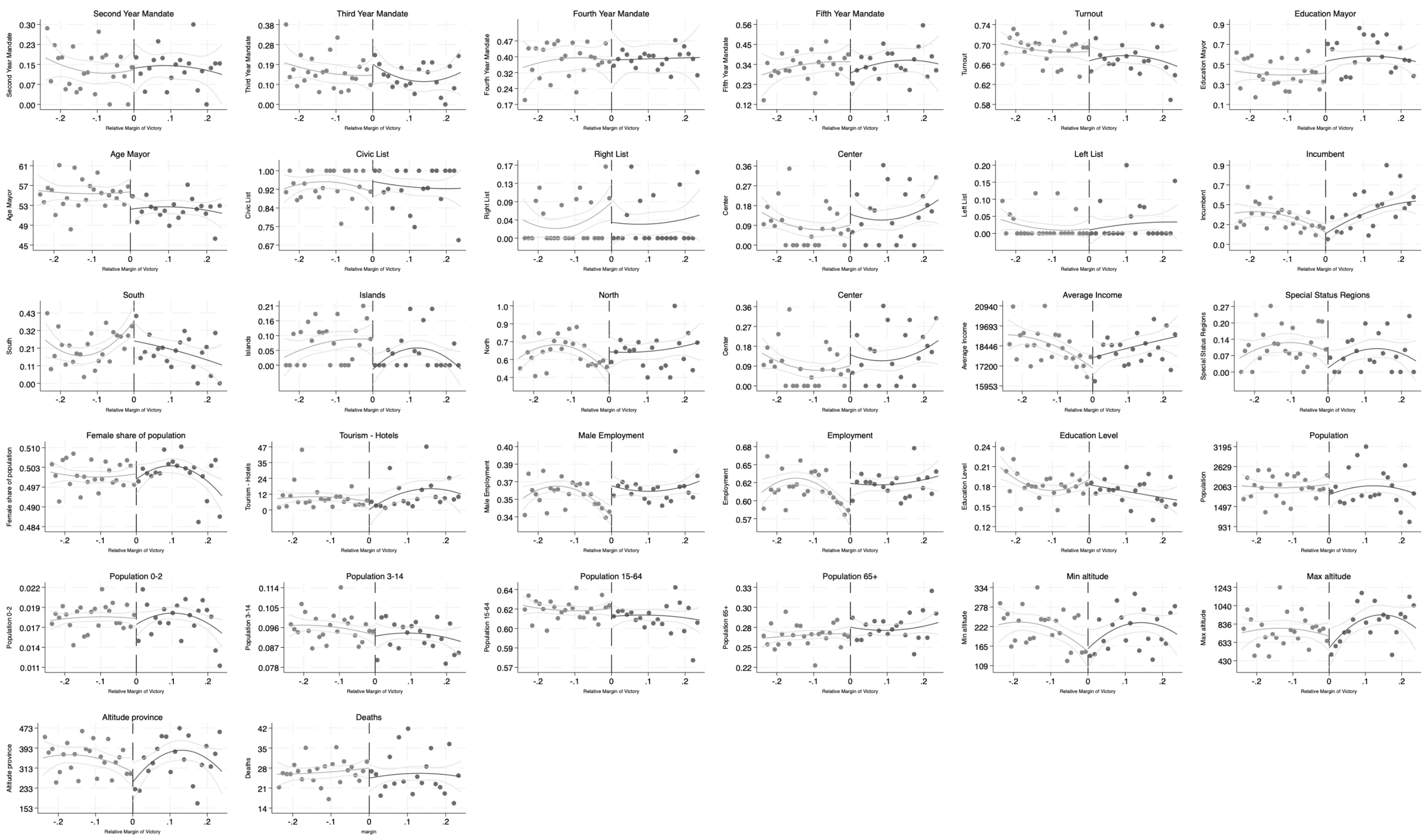

Notes: The figure plots the binned averages of 32 variables for mayor, election and municipalities characteristics against the relative margin, together with the quadratic polynomial fit on both sides of the 0 relative margin cut-off and the 95% confidence intervals. These plots refer only to the after Covid-19 period (2022-2023). Observations on the right hand side of the cut-off refer to those municipalities in which the mayor is a woman, while those on the left hand side refer to municipalities in which the mayor is a man.

Figure A.6: Additional bandwidths

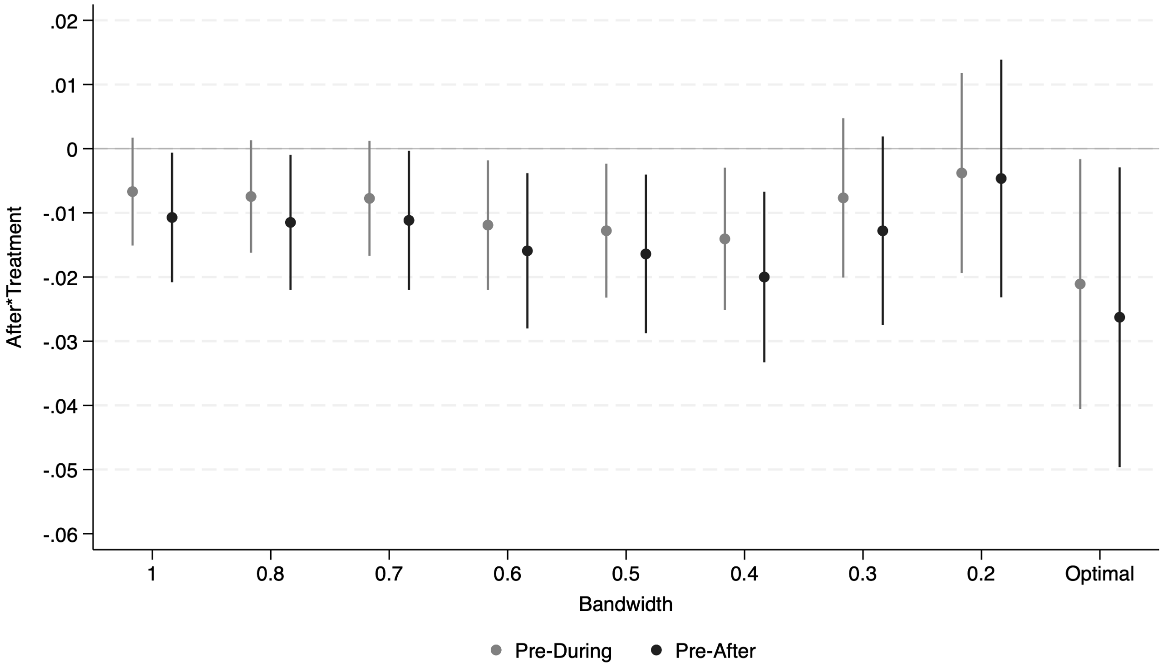

Notes: This graph shows After*Treatment coefficients for the pre-during and pre-after Covid-19 period at different bandwidths. The outcome variable is spending on childcare in shares. 95% confidence intervals are included. Optimal bandwidths are computed at the optimal bandwidth according to Calonico et al. (2014). In all specifications, we control for municipality, election, and mayor's characteristics, which are not consistently continuous in the three periods and for covariates that are not similar across periods. Therefore, in pre-during period, we control for turnout, special statute regions, average income, altitude max, altitude province, south, islands, north, employment rate, male employment rate, second year of mandate, third year of mandate, fourth year of mandate, fifth year of mandate, share of population aged 0-2, share of population aged 15-64, share of population aged 65+, share of female population, education of the mayor, age of the mayor and average education level of the municipality, and in pre-after, we control for second year of mandate, third year of mandate, fourth year of mandate, fifth year of mandate, turnout, education of the mayor, age of the mayor, center list, south, islands, north, special statute regions, average income, share of female population, share of population aged 0-2, share of population aged 3-14, share of population aged 15-64, share of population aged 65+, employment rate, male employment rate, altitude province and average education level of the municipality.

Figure A.7: Spending on childcare (in shares) by year

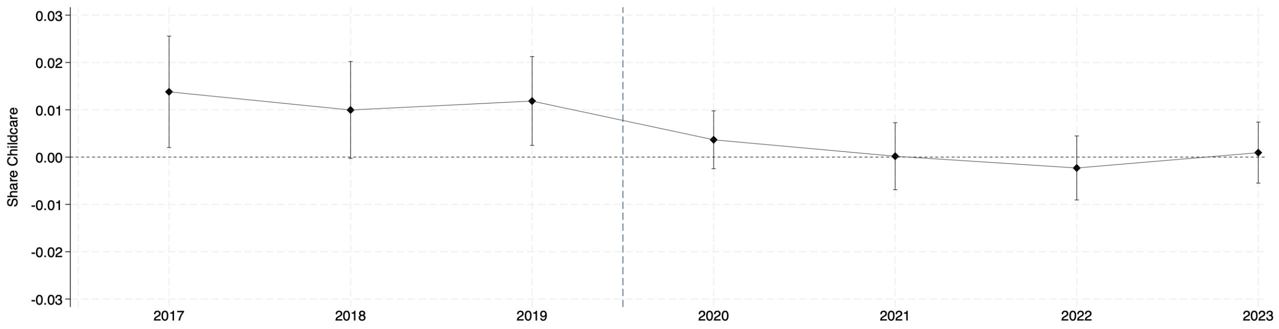

Notes: This graph shows yearly regression discontinuity coefficients for the difference in spending on childcare (in shares) between female and male mayors. 95% confidence intervals are included.

Figure A.8: Correlation between gender and ideology in larger municipalities

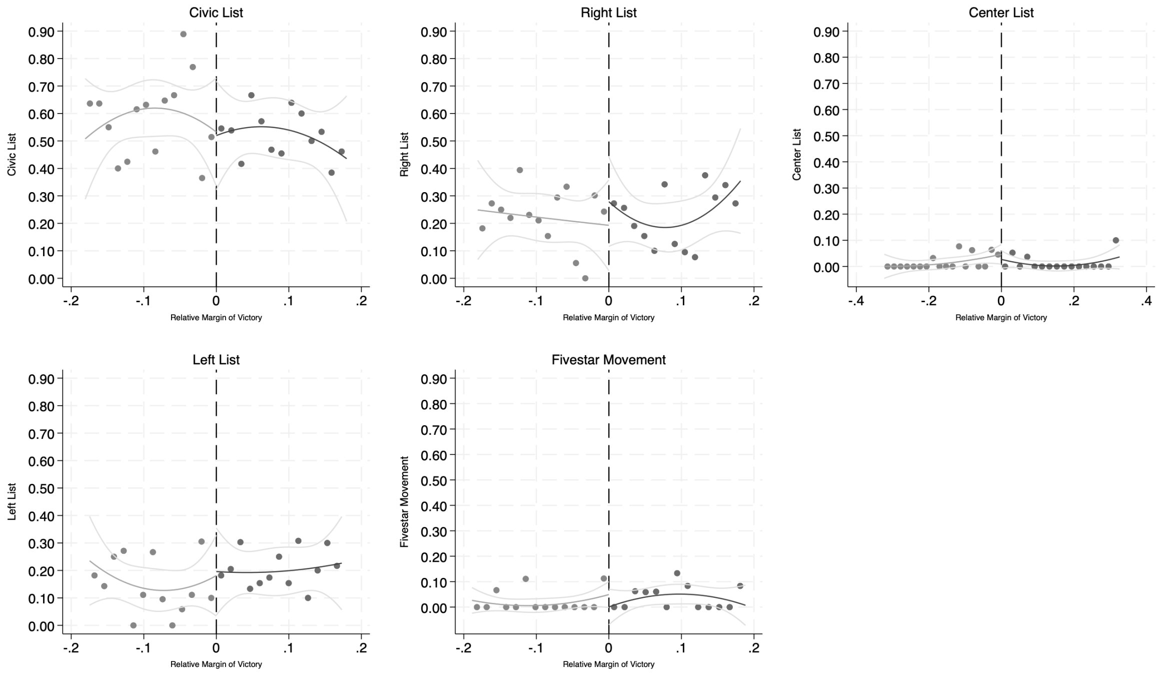

Notes: This figure plots regression discontinuity estimates for the likelihood of belonging to a given party conditional on mayor gender in municipalities with more than 5,000 inhabitants in which occurred a close mixed gender race between 2016 and 2022. Observations on the right-hand side (i.e., positive relative margin of victory) refer to those municipalities in which the mayor is a woman, while those on the left-hand side refer to municipalities in which the mayor is a man. The 95% confidence intervals are displayed in the figure.

Table A.1: Mayor's, election and municipality characteristics

Period	Pre	During	After
Column	(1)	(2)	(3)
Second Year Mandate	-0.012	0.004	0.037
	(0.04)	(0.03)	(0.04)
Third Year Mandate	0.024	-0.046*	0.025
	(0.03)	(0.03)	(0.04)
Fourth Year Mand	-0.012	0.010	-0.013
	(0.04)	(0.02)	(0.04)
Fifth Year Mand	0.000	0.032	-0.049
	(.)	(0.03)	(0.04)
Turnout	-0.030	-0.016	-0.004
	(0.03)	(0.01)	(0.02)
University Degree Mayor	0.092	0.166***	0.169**
	(0.14)	(0.05)	(0.08)
Age Mayor	-3.647	-3.394***	-2.654
	(3.24)	(1.09)	(1.82)
Civic List	-0.029	-0.017	0.018
	(0.07)	(0.03)	(0.04)
Right List	0.023	-0.009	-0.031
	(0.06)	(0.02)	(0.03)
Left List	0.015	0.019	0.010
	(0.04)	(0.02)	(0.02)
Center List	-0.009	0.006	0.003
	(0.02)	(0.01)	(0.01)
South	0.262**	0.011	-0.026
	(0.12)	(0.04)	(0.07)
Islands	-0.298***	-0.020	-0.072**
	(0.10)	(0.02)	(0.03)
North	0.065	-0.022	0.055
	(0.15)	(0.05)	(0.08)
Center	-0.029	0.032	0.042
	(0.08)	(0.03)	(0.05)
Average Income	-766.875	-467.143	399.441
	(1208.24)	(391.44)	(610.63)
Special Statute Regions	-0.251**	-0.026	-0.056
	(0.12)	(0.02)	(0.04)
Female Population	-0.014***	0.001	0.003
	(0.00)	(0.00)	(0.00)
Tourism	-2.490	-0.864	-1.749
	(2.24)	(2.81)	(4.08)
Employment Rate	0.011	-0.006	0.022*
	(0.02)	(0.01)	(0.01)
Male Employment Rate	0.014	-0.001	0.012**
	(0.01)	(0.00)	(0.01)
Education Level (share)	-0.018	-0.005	0.007
	(0.02)	(0.01)	(0.01)
Population	282.338	-80.596	-99.376
	(382.77)	(133.55)	(221.38)
Population Aged 02 (share)	0.001	0.000	0.000
	(0.00)	(0.00)	(0.00)
Population Aged 3-14 (share)	0.008	-0.001	0.000
	(0.01)	(0.00)	(0.00)
Population Aged 1564 (share)	-0.006	-0.005	-0.004
	(0.01)	(0.00)	(0.01)
Population Aged 65 (share)	-0.003	0.006	0.003
	(0.01)	(0.01)	(0.01)
Incumbent	0.019	0.001	-0.029
	(0.12)	(0.04)	(0.07)
Min Altitude	57.205	-2.247	19.883
	(64.79)	(19.63)	(28.31)
Max Altitude	174.452	50.011	-13.323
	(210.16)	(73.61)	(114.39)
Altitude Province	170.724**	11.485	6.654
	(85.14)	(26.97)	(41.30)
Deaths	0.999	-1.141	-2.366
	(4.63)	(1.79)	(2.74)

Notes: The table shows 32 separate RD regressions repeated over three times periods (i.e. pre Covid-19 (2016-2019) in column 1, during Covid-19 (2020-2021) in column 2 and after Covid-19 (2022-2023) in column 3. The running variable is the relative margin, while the 32 different outcomes, reported in the table, are municipal, mayor and election characteristics. All the regressions are computed at the optimal bandwidth according to Calonico et al. (2014) for the three periods separately and standard errors are clustered at municipal level. *p<0.10, **p<0.05, ***p<0.01.

Table A.2: Robustness checks

	Balanced data		Second order polynomial		No Covid-19 elections		No controls	
	Pre-During	Pre-After	Pre-During	Pre-After	Pre-During	Pre-After	Pre-During	Pre-After
Column	(1)	(2)	(3)	(4)	(5)	(6)	(7)	(8)
Female	0.027**	0.020*	0.019*	0.018*	0.018*	0.018*	0.017*	0.016*
	(0.01)	(0.01)	(0.01)	(0.01)	(0.01)	(0.01)	(0.01)	(0.01)
Post	0.013*	0.025**	0.016**	0.020**	0.015**	0.024***	0.010	0.013**
	(0.01)	(0.01)	(0.01)	(0.01)	(0.01)	(0.01)	(0.01)	(0.01)
Post*Female	-0.017*	-0.020	-0.021**	-0.026**	-0.021**	-0.030**	-0.021**	-0.023*
	(0.01)	(0.02)	(0.01)	(0.01)	(0.01)	(0.01)	(0.01)	(0.01)
Female+Post*Female	0.010	-0.000	-0.002	-0.008	-0.003	-0.012	-0.004	-0.007
Post+Post*Female	-0.005	0.005	-0.005	-0.007	-0.006	-0.006	-0.011	-0.010
Bandwidth	0.110	0.120	0.110	0.120	0.110	0.120	0.110	0.120
R^2	0.416	0.433	0.203	0.204	0.200	0.202	0.011	0.018
N	227	212	855	843	827	670	865	852

Notes: The table compares RD estimates over time, implementing some robustness checks. The outcome is always spending on childcare (in shares). In columns 1-2, we run our analysis on a balanced dataset including only municipalities for which we have at least one observation for each of the three periods. In columns 3 and 4, we include second-order polynomials in our RD model. In columns 5 and 6, we drop elections that took place after 2019, and in columns 7 and 8, we run the analysis without including controls. In columns 1, 3, 5, and 7, data from 2016 to 2021 are included, allowing for a comparison of gender differences before Covid-19 (2016-2019) with those during the pandemic (2020-2021). Columns 2, 4, 6, and 8 include data from the pre pandemic period (2016-2019) and the recovery period (2022-2023), allowing a comparison of gender differences before Covid-19 with those following the end of the Covid-19 emergency. $Female$ is a dummy equal to 1 if the mayor is female and 0 otherwise; $Post$ is a dummy that identifies time. In columns 1, 3, 5, and 7, it is equal to 1 if the observation is in the during-Covid-19 period and 0 for the pre-Covid-19 period. In columns 2, 4, 6, and 8, it is equal to 1 if the observation is in the after-Covid-19 period and 0 for the pre-Covid-19 period. $Post * Treatment$ is the variable of interest. All results are carried out at optimal bandwidth according to Calonico et al. (2014) of our main specifications. In columns 1-6, we control for municipality, election, and mayor's characteristics. In columns 1, 3, and 5, we control for turnout, special statute regions, average income, altitude max, altitude province, south, islands, north, employment rate, male employment rate, second year of mandate, third year of mandate, fourth year of mandate, fifth year of mandate, share of population aged 0-2, share of population aged 15-64, share of population aged 65+, share of female population, education of the mayor, age of the mayor, and average education level of the municipality. In columns 2, 4, and 6, we control for second year of mandate, third year of mandate, fourth year of mandate, fifth year of mandate, turnout, education of the mayor, age of the mayor, center list, south, islands, north, special statute regions, average income, share of female population, share of population aged 0-2, share of population aged 3-14, share of population aged 15-64, share of population aged 65+, average education level of the municipality, employment rate, male employment rate, and altitude province. Standard errors, reported in parentheses, are clustered at the municipal level. *p<0.10, **p<0.05, ***p<0.01.

Table A.3: Dropping one year at the time

Panel A: Pre-During					
Without	2017	2018	2019	2020	2021
Share Childcare					
Female	0.015	0.021**	0.022*	0.020**	0.018*
	(0.01)	(0.01)	(0.01)	(0.01)	(0.01)
Post	0.015**	0.018***	0.015*	0.018***	0.014**
	(0.01)	(0.01)	(0.01)	(0.01)	(0.01)
Post*Female	-0.017*	-0.024**	-0.024*	-0.022**	-0.020**
	(0.01)	(0.01)	(0.01)	(0.01)	(0.01)
Female+Post*Female	-0.002	-0.002	-0.002	-0.002	-0.002
Post+Post*Female	-0.001	-0.006	-0.009	-0.004	-0.006
Bandwidth	0.11	0.11	0.11	0.11	0.11
R^2	0.208	0.235	0.210	0.178	0.193
N	784	748	730	552	606

Panel B: Pre-After					
Without	2017	2018	2019	2022	2023
Share Childcare					
Female	0.013	0.020**	0.023*	0.018*	0.019*
	(0.01)	(0.01)	(0.01)	(0.01)	(0.01)
Post	0.019**	0.022***	0.017*	0.020**	0.019**
	(0.01)	(0.01)	(0.01)	(0.01)	(0.01)
Post*Female	-0.021*	-0.029**	-0.030**	-0.025**	-0.027**
	(0.01)	(0.01)	(0.01)	(0.01)	(0.01)
Female+Post*Female	-0.008	-0.009	-0.008	-0.007	-0.009
Post+Post*Female	-0.002	-0.007	-0.014	-0.005	-0.008
Bandwidth	0.12	0.12	0.12	0.12	0.12
R^2	0.208	0.249	0.215	0.178	0.186
N	772	736	716	568	580

Notes: The table compares RD estimates over time dropping one year at the time. The outcome variable is spending on childcare (in shares). Panel A includes data from 2016 to 2021, allowing for a comparison of gender differences before Covid-19 (2016-2019) with those during the pandemic (2020-2021) and in each column one year is dropped from the analysis: year 2017 is dropped in column 1, 2018 in column 2, 2019 in column 3, 2020 in column 4 and 2021 in column 5. Panel B includes data from the pre-pandemic period (2016-2019) and the recovery period (2022-2023), allowing for a comparison of gender differences before Covid-19 with those following the end of the Covid-19 emergency and in each column one year is dropped from the analysis: year 2017 is dropped in column 1, 2018 in column 2, 2019 in column 3, 2022 in column 4 and 2023 in column 5. $Female$ is a dummy equal to 1 if the mayor is female and equal to 0 otherwise; $Post$ is a dummy that identifies time. $Post * Treatment$ is the variable of interest. Results for the pre-during analysis and the pre-after analysis are carried out at optimal bandwidth according to Calonico et al. (2014) of our main specifications. In all specifications, we control for municipality, election, and mayor's characteristics, which are not consistently continuous in the three periods and for covariates that are not similar across periods. Therefore, in Panel A, we control for turnout, special statute regions, average income, altitude max, altitude province, south, islands, north, employment rate, male employment rate, second year of mandate, third year of mandate, fourth year of mandate, fifth year of mandate, share of population aged 0-2, share of population aged 15-64, share of population aged 65+, share of female population, education of the mayor, age of the mayor and average education level of the municipality, and in Panel B, we control for second year of mandate, third year of mandate, fourth year of mandate, fifth year of mandate, turnout, education of the mayor, age of the mayor, center list, south, islands, north, special statute regions, average income, share of female population, share of population aged 0-2, share of population aged 3-14, share of population aged 15-64, share of population aged 65+, average education level of the municipality, employment rate, male employment rate, altitude province. Standard errors, reported in parentheses, are clustered at the municipal level. *p<0.10, **p<0.05, ***p<0.01.

Table A.4: Current spending on childcare (in shares), pooling during and after Covid-19 years

Period	Pre(2016-2019)-Post(2020-2023)		
Column	(1)	(2)	(3)
Share Childcare			
Female	0.010**	0.015***	0.017*
	(0.00)	(0.01)	(0.01)
Post	0.005	0.009**	0.017**
	(0.00)	(0.00)	(0.01)
Post*Female	-0.009*	-0.014**	-0.025**
	(0.00)	(0.01)	(0.01)
Female+Post*Female	0.001	0.000	-0.007
Post+Post*Female	-0.004	-0.006	-0.007
Bandwidth	1.00	0.50	0.10
R^2	0.174	0.184	0.233
N	4763	3831	1231

Notes: The table compares RD estimates over time. The outcome variable is spending on childcare (in shares). The results refer tot the period pre-post Covid-19 period. *Female* is a dummy equal to 1 if the mayor is female and equal to 0 otherwise; *Post* is equal to 1 if the observation is in the post Covid-19 period and 0 for the pre-Covid-19 period. $Post * Treatment$ is the variable of interest. Results are carried out at three different bandwidths: whole sample (column 1), 0.5 (column 2), and optimal bandwidth according to Calonico et al. (2014) (column 3). In all specifications, we control for municipality, election, and mayor's characteristics, which are not consistently continuous in the two periods and for covariates that are not similar across periods. In particular we control for second year of mandate, third year of mandate, fifth year of mandate, turnout, education of the mayor, age of the mayor, special statute regions, center list, south, north, islands, average income, employment rate, male employment rate, average education level of the municipality, share of population aged 0-2, share of population aged 15-64, share of population aged 65+, max altitude, altitude province, share of female population and incumbent. Standard errors, reported in parentheses, are clustered at the municipal level. *p<0.10, **p<0.05, ***p<0.01.

Table A.5: Current spending on childcare (in shares) over time: three time periods

Column	(1)	(2)	(3)
Share Childcare			
woman	0.003	0.002	-0.003
	(0.00)	(0.00)	(0.01)
t1	-0.004	-0.008**	-0.016**
	(0.00)	(0.00)	(0.01)
t2	0.003*	0.003*	0.005*
	(0.00)	(0.00)	(0.00)
Female*t1	0.007	0.013**	0.021**
	(0.00)	(0.01)	(0.01)
Female*t2	-0.003	-0.003	-0.005
	(0.00)	(0.00)	(0.00)
-t1-Female*t1	-0.003	-0.005	-0.005
Bandwidth	1.000	0.500	0.110
R^2	0.175	0.183	0.224
N	4763	3831	1381

Notes: The table compares RD estimates over time. The outcome variable is share spending on childcare. *Female* is a dummy equal to 1 if the mayor is female and equal to 0 otherwise; $t1$ and $t2$ are dummies that identify time. $t1$ equals 1 before 2020 and 0 otherwise, while $t2$, equals 1 for the post Covid-19 years (after 2021) and 0 otherwise. The coefficient of the interaction term $Female * t1$ captures the difference between the gender gap before the pandemic and the gender gap in the Covid-19 years. Results are carried out at three different bandwidths: whole sample (column 1), 0.5 (column 2), and optimal bandwidth according to Calonico et al. (2014) (column 3). In all specifications, we control for municipality, election, and mayor's characteristics, which are not consistently continuous in the three periods and for covariates that are not similar across periods. Therefore, we control for second year of mandate, third year of mandate, fourth year of mandate, fifth year of mandate, turnout, education of the mayor, age of the mayor, center list, south, islands, north, special statute regions, average income, share of female population, share of population aged 0-2, share of population aged 3-14, share of population aged 15-64, share of population aged 65+, average education level of the municipality, employment rate, male employment rate, altitude province and max altitude. Standard errors, reported in parentheses, are clustered at the municipal level. *p<0.10, **p<0.05, ***p<0.01.

Table A.6: Heterogeneity by share of female members in the local government

Share of female members	Below Median			Above Median		
Column	(1)	(2)	(3)	(4)	(5)	(6)
Share Childcare						
Female	0.011**	0.013*	0.010	0.011	0.020**	-0.005
	(0.01)	(0.01)	(0.01)	(0.01)	(0.01)	(0.01)
Post	0.011**	0.015***	0.014*	0.004	0.007	0.016
	(0.00)	(0.01)	(0.01)	(0.00)	(0.01)	(0.01)
Post*Female	-0.011*	-0.015*	-0.013	-0.014	-0.022**	-0.003
	(0.01)	(0.01)	(0.01)	(0.01)	(0.01)	(0.01)
Female+Post*Female	-0.000	-0.002	-0.004	-0.003	-0.002	-0.008
Post+Post*Female	-0.000	0.000	0.001	-0.010	-0.015*	0.013*
Bandwidth	1.00	0.50	0.21	1.00	0.50	0.11
R^2	0.205	0.229	0.256	0.145	0.153	0.212
N	1314	1043	656	1527	1238	414

Notes: The table compares RD estimates over time for municipalities with a share of female members in the local government below the median (columns 1-3) and above the median (columns 4-6). The analysis is carried out only on the period pre-after Covid-19. In all columns the dependent variable is spending on childcare (in shares). *Female* is a dummy equal to 1 if the mayor is female and equal to 0 otherwise; *Post* is a dummy equal to 1 if the observation is in the after Covid-19 period and 0 for the pre Covid-19 period. Observations from the years 2020 and 2021 are not included in this analysis. $Post * Treatment$ is the variable of interest and it identifies female vs male after Covid-19, compared to female vs male before Covid-19. Results are carried out at three different bandwidths: whole sample (columns 1 and 4), 0.5 (columns 2 and 5), and optimal bandwidth according to Calonico et al. (2014) (columns 3 and 6). In all specifications, we control for municipality, election, and mayors' characteristics, which are not consistently continuous in the three periods and for covariates that are not similar across periods, namely second year of mandate, third year of mandate, fourth year of mandate, fifth year of mandate, turnout, education of the mayor, age of the mayor, center list, south, islands, north, special statute regions, average income, share of female population, share of population aged 0-2, share of population aged 3-14, share of population aged 15-64, share of population aged 65+, average education level of the municipality, employment rate, male employment rate, altitude province. Standard errors, reported in parentheses, are clustered at the municipal level. *p<0.10, **p<0.05, ***p<0.01.

Table A.7: Other spending categories and entries

Panel A: Pre-During													
Share	Youth	Law & Order	Rights	Health	Transurban	Institution	Debt	Economics	Tot. current spending (log)	Entries from Taxes	Extra Taxes Entries	Entries from Transfers	Tot. current entries (log)
Column	(1)	(2)	(3)	(4)	(5)	(6)	(7)	(8)	(9)	(10)	(11)	(12)	(13)
Female	-0.020	0.001	0.003	0.004	0.001	-0.007	0.006	-0.005	0.231	0.012	0.020	-0.019	0.137
	(0.02)	(0.01)	(0.02)	(0.02)	(0.02)	(0.03)	(0.01)	(0.01)	(0.16)	(0.04)	(0.03)	(0.03)	(0.15)
Post	-0.022*	-0.005	0.020*	0.013	-0.004	-0.023	-0.002	0.006	0.376***	-0.036	-0.009	0.046**	0.347***
	(0.01)	(0.01)	(0.01)	(0.01)	(0.02)	(0.02)	(0.00)	(0.01)	(0.11)	(0.02)	(0.02)	(0.02)	(0.11)
Post*Female	0.014	-0.001	0.003	0.007	-0.009	0.018	0.002	0.008	-0.318**	-0.017	-0.020	0.023	-0.247*
	(0.01)	(0.01)	(0.02)	(0.02)	(0.02)	(0.03)	(0.01)	(0.01)	(0.15)	(0.04)	(0.03)	(0.03)	(0.15)
Female+Post*Female	-0.006	-0.001	0.006	0.010	-0.008	0.011	0.007*	0.004	-0.087	-0.005	-0.000	0.004	-0.110
Post+Post*Female	-0.008	-0.007	0.022 **	0.019	-0.013	-0.005	-0.000	0.014***	0.058	-0.053 *	-0.029	0.068***	0.100
Bandwidth	0.17	0.24	0.27	0.19	0.20	0.15	0.16	0.24	0.18	0.14	0.20	0.12	0.21
R^2	0.327	0.148	0.194	0.216	0.259	0.229	0.085	0.099	0.407	0.510	0.158	0.735	0.399
N	1240	1589	1687	1316	1392	1091	1161	1589	1288	1046	1401	937	1449

Panel B: Pre-After													
Share	Youth	Law & Order	Rights	Health	Transurban	Institution	Debt	Economics	Tot. current spending (log)	Entries from Taxes	Extra Taxes Entries	Entries from Transfers	Tot. current entries (log)
Column	(1)	(2)	(3)	(4)	(5)	(6)	(7)	(8)	(9)	(10)	(11)	(12)	(13)
Female	-0.024	0.000	-0.002	-0.002	-0.004	0.012	0.008	-0.006	0.164	0.034	0.007	-0.017	0.124
	(0.02)	(0.01)	(0.02)	(0.02)	(0.02)	(0.03)	(0.01)	(0.01)	(0.16)	(0.04)	(0.03)	(0.03)	(0.16)
Post	-0.008	-0.008	0.017	0.002	-0.038**	-0.017	0.017***	0.010	0.571***	-0.027	0.011	0.036	0.504***
	(0.01)	(0.01)	(0.01)	(0.02)	(0.02)	(0.03)	(0.01)	(0.01)	(0.13)	(0.03)	(0.02)	(0.03)	(0.13)
Post*Female	0.010	-0.001	-0.003	-0.002	0.008	0.013	-0.003	0.006	-0.350*	-0.018	-0.010	-0.014	-0.306
	(0.02)	(0.01)	(0.02)	(0.03)	(0.03)	(0.04)	(0.01)	(0.01)	(0.19)	(0.05)	(0.04)	(0.04)	(0.19)
Female+Post*Female	-0.014*	-0.001	-0.005	-0.004	0.004	0.025	0.004	0.001	-0.186*	0.016	-0.003	-0.032	-0.182*
Post+Post*Female	0.002	-0.009	0.014	0.000	-0.030	-0.004	0.013**	0.016***	0.221	-0.045	0.001	0.022	0.198
Mean depvar	0.098	0.032	0.052	0.037	0.291	0.418	0.029	0.016	14.183	0.613	0.193	0.199	14.288
Bandwidth	0.17	0.24	0.25	0.17	0.22	0.15	0.20	0.22	0.19	0.15	0.19	0.12	0.19
R^2	0.306	0.143	0.202	0.201	0.252	0.187	0.210	0.117	0.366	0.520	0.137	0.728	0.350
N	1202	1567	1580	1211	1429	1051	1350	1440	1288	1076	1300	912	1338

Notes: The table compares RD estimates over time. The outcome variables are spending for youth, spending for law and order, spending for rights, spending for health, spending for transports and urban planning, spending for institutions, spending for debt, spending for support to the local economy, all in shares, total current spending (log), entries from taxes, extra taxes entries, entries from transfers, all in shares and total current entries (log). Panel A includes data from 2014 to 2021, allowing for a comparison of gender differences before Covid-19 (2014-2019) with those during the pandemic (2020-2021). Panel B includes data from the pre-pandemic period (2014-2019) and the recovery period (2022-2023), allowing for a comparison of gender differences before Covid-19 with those following the end of the Covid-19 emergency. $Female$ is a dummy equal to 1 if the mayor is female and equal to 0 otherwise; $Post$ is a dummy that identifies time. $Post * Treatment$ is the variable of interest. Results for the pre-during and pre-after analyses are carried out at the optimal bandwidth according to Calonico et al. (2014). In all specifications, we control for municipality, election, and mayor's characteristics, which are not consistently continuous in the three periods and for covariates that are not similar across periods. Therefore, in Panel A, we control for turnout, special statute regions, average income, altitude max, altitude province, south, islands, north, employment rate, male employment rate, second year of mandate, third year of mandate, fourth year of mandate, fifth year of mandate, share of population aged 0-2, share of population aged 15-64, share of population aged 65+, share of female population, education of the mayor, age of the mayor and average education level of the municipality. In Panel B, we control for second year of mandate, third year of mandate, fourth year of mandate, fifth year of mandate, turnout, education of the mayor, age of the mayor, center list, south, islands, north, special statute regions, average income, share of female population, share of population aged 0-2, share of population aged 3-14, share of population aged 15-64, share of population aged 65+, average education level of the municipality, employment rate, male employment rate, altitude province. Standard errors, reported in parentheses, are clustered at the municipal level. *p<0.10, **p<0.05, ***p<0.01.
